# Concentration-Dependent Activity of Hydromethylthionine on Cognitive Decline and Brain Atrophy in Mild to Moderate Alzheimer’s Disease

**DOI:** 10.3233/JAD-190772

**Published:** 2019-11-26

**Authors:** Bjoern O. Schelter, Helen Shiells, Thomas C. Baddeley, Christopher M. Rubino, Harish Ganesan, Jeffrey Hammel, Vesna Vuksanovic, Roger T. Staff, Alison D. Murray, Luc Bracoud, Gernot Riedel, Serge Gauthier, Jianping Jia, Peter Bentham, Karin Kook, John M.D. Storey, Charles R. Harrington, Claude M. Wischik

**Affiliations:** a TauRx Therapeutics Ltd., Singapore, Singapore; b Institute for Complex Systems and Mathematical Biology, University of Aberdeen, Aberdeen, UK; c Department of Chemistry, University of Aberdeen, Aberdeen, UK; d Institute of Clinical Pharmacodynamics, Schenectady, NY, USA; e Aberdeen Biomedical Imaging Center, University of Aberdeen, Foresterhill, Aberdeen, UK; f Aberdeen Royal Infirmary, NHS Grampian, Aberdeen, UK; g Bioclinica, Lyon, France; h School of Medicine, Medical Sciences and Nutrition, University of Aberdeen, Foresterhill, Aberdeen, UK; i McGill Centre for Studies in Aging, Alzheimer’s Disease Research Unit, and Douglas Mental Health University Institute, Montreal, QC, Canada; j Innovation Center for Neurological Disorders, Neurology Department, Xuanwu Hospital, Capital Medical University, Beijing, China; k Salamandra LLC, Bethesda, MD, USA

**Keywords:** Acetylcholinesterase inhibitor, Alzheimer’s disease, clinical trials, drug interaction, leucomethylthioninium, population pharmacokinetics, hydromethylthionine

## Abstract

**Background:**

Although hydromethylthionine is a potent tau aggregation inhibitor, no difference was found in either of two Phase III trials in mild to moderate Alzheimer’s disease (AD) comparing doses in the range 150–250 mg/day with 8 mg/day intended as a control.

**Objective:**

To determine how drug exposure is related to treatment response.

**Methods:**

A sensitive plasma assay for the drug was used in a population pharmacokinetic analysis of samples from 1,162 of the 1,686 patients who participated in either of the Phase III trials with available samples and efficacy outcome data.

**Results:**

There are steep concentration-response relationships for steady state plasma levels in the range 0.3–0.8 ng/ml at the 8 mg/day dose. Using a threshold based on the lower limit of quantitation of the assay on Day 1, there are highly significant differences in cognitive decline and brain atrophy in patients with above threshold plasma levels, both for monotherapy and add-on therapy, but with effect sizes reduced by half as add-on. Plasma concentrations in the range 4–21 ng/ml produced by the high doses are not associated with any additional benefit.

**Conclusions:**

Hydromethylthionine has pharmacological activity on brain structure and function at the 8 mg/day dose as monotherapy or as add-on to symptomatic treatments. This combined with a plateau at higher doses is consistent with the lack of dose-response seen in the Phase III trials. Treatment benefit is predicted to be maximal at 16 mg/day as monotherapy. A placebo-controlled trial in mild/moderate AD is now ongoing to confirm efficacy at this dose.

## INTRODUCTION

Alzheimer’s disease (AD) is an irreversible neurodegenerative disease affecting as many as 1 in 5 of the population over the age of 65 years. The only approved treatments are symptomatic. The most widely used of these are the acetylcholinesterase inhibitors (AChEIs) which work by chronically increasing the levels of acetylcholine in the synaptic cleft. In experimental models, cholinergic function is associated primarily with selective attention [1–3], and is not particularly sensitive to more broadly based measures of functional impairment/improvement (reviewed in [4, 5]). Similar considerations apply to memantine, which also modulates brain function in a non-specific manner [6, 7]. Although the small therapeutic benefit of these treatments persists over time, patients continue to decline at the untreated rate [8], with fewer than 30% of patients continuing on AChEIs 12 months after initiation [9–11]. A substantial proportion of AD patients are not treated, ∼44% in the US [12] and ∼77% in UK [13]. In France, reimbursement for these drugs has been withdrawn because of “insufficient medical benefit and dangerousness because of side effects” [14]. Hence it is agreed generally that a major unmet medical need exists to develop a treatment able to slow the progression of AD. A Lancet Neurology Commission report noted that “ ...   no treatment is yet available to halt or reverse the underlying pathology of established AD. Indeed, an effective therapy for AD is perhaps the greatest unmet need facing modern medicine” [15].

From 2002 to 2012, there were 289 clinical trials at Phase II or Phase III, with an overall failure rate of 99.6% [16], and a further 19 trial failures since 2012 targeting various aspects of pathological processing of amyloid-β [17]. There is now increasing recognition of the importance of tau aggregation pathology as an important substrate of clinical dementia and as a target for therapy [18]. The most advanced late-stage program targeting tau aggregation currently in development is based on leuco-methylthioninium bis(hydromethanesulphonate) (LMTM) [19]. LMTM has recently been assigned the International Nonproprietary Name “hydromethylthionine”, recognizing it as chemically and pharmacologically distinct from methylthioninium chloride (MTC, methylene blue). The methylthioninium (MT) moiety can exist in oxidized (MT^+^) and reduced (LMT) forms. LMTM is a stabilized dihydromesylate salt of LMT which has more favorable pharmaceutical properties than the oxidized MT^+^ form administered as MTC [19, 20]. We have retained the LMTM abbreviation in the present paper as it facilitates technical discussion of the distinctive properties of LMT. LMT, and not MT^+^, is the active species that blocks tau aggregation *in vitro* acting at a tau:LMT molar ratio of 1 : 0.1 [21]. In earlier studies, the MT moiety was also found to reverse the proteolytic stability of tangle filaments isolated from AD brain tissues at a similar molar ratio [19, 22]. It is therefore a potent tau aggregation inhibitor with a site of action within the proteolytically stable core tau unit of the tangle filament [23–25]. LMT also blocks tau aggregation in cell-based assays [19, 21] and reduces tau aggregation pathology and associated behavioral deficits in tau transgenic mouse models *in vivo* at a dose of 9 mg/kg/day [26]. This corresponds approximately to a human dose of 8–16 mg/day in terms of plasma C_max_ considering that the half-life in mice is 4 h compared with 37 h in elderly humans.

The MT moiety also has a range of other properties that affect cellular metabolism. It has been known for some time that it enhances mitochondrial activity at low concentrations (10–100 nM) by acting as a supplementary electron carrier in the electron transfer chain [27, 28]. It is able to induce mitochondrial biogenesis and to activate Nrf2-mediated oxidative stress response elements *in vivo* [29]. Other potentially beneficial activities include neuroprotective effects in the brain by inhibiting microglial activation [30] and enhancing autophagy at the 10–20 nM concentration range [30, 31]. In a more recent study in a tau transgenic mouse model for AD, LMTM at doses of 5 and 15 mg/kg/day was found to increase acetylcholine levels in hippocampus, restore choline acetyltransferase activity in basal forebrain, reverse impairment in glutamate release from brain synaptosomes and increase Complex IV activity in brain mitochondria [32]. Therefore, in addition to prevention and dissolution of AD tau aggregates [19, 22], LMTM has numerous complementary actions which address many of the pathways currently advocated as having potential for the treatment of AD [33, 34].

Given these potentially useful pharmacological properties, it was surprising that LMTM failed to show any difference in two Phase III trials in which patients were randomized to compare doses in the range 150–250 mg/day with a low dose (8 mg/day) that was intended as a control to mask the variable discoloration of urine that can occur on exposure to air following excretion [35, 36]. The high doses were selected on the basis of an earlier placebo-controlled dose-finding Phase II study which showed that the minimum effective dose is 138 mg/day for MTC, and early comparative pharmacokinetic (PK) studies showing similar plasma levels of total MT measured after acid extraction of samples [37]. However, we have found that this assay is dominated by an acid-labile inactive conjugate of LMT in plasma and this is not distinguished from the active parent form of the drug. We have developed a sensitive assay which can measure parent MT levels in plasma, and which has been found to be reliable and accurate in five Phase I studies and 14 preclinical studies.

We have used this assay to measure blood samples collected from patients participating in the two Phase III trials to determine the extent to which drug exposure determines treatment response on clinical and MRI volumetric endpoints. If there is any concentration-response relationship, then the further objectives were to explore how co-medication status with treatments approved for AD and drug exposure interact in terms of plasma levels and treatment response, and to determine the most suitable dose for testing in a further randomized placebo-controlled trial.

## MATERIALS AND METHODS

### Study patients

Pharmacokinetic analyses were undertaken using plasma concentration data from patients who participated in either of two completed Phase III trials which have been described previously [35, 36]. In brief, the first (TRx-237-015) was a 15-month study which recruited 890 patients from 115 sites across 16 countries in EU, North America, Asia, and Russia. Patients had to be <90 years of age and have a diagnosis of mild to moderate probable AD according to National Institute of Aging (NIA) and Alzheimer’s Association (AA) criteria [38] and a Mini-Mental State Examination (MMSE) score of 14–26 inclusive and with a Clinical Dementia Rating (CDR) total score of 1 or 2. The second study (TRx-237-005) was an 18-month trial which recruited 800 patients from 108 sites in Canada, United States, Australia, and Europe. Patients had to meet the same age and diagnosis criteria, except that they were required to have a MMSE score of 20–26 inclusive and a CDR total score of 0.5 or 1.0. Concomitant use of AChEIs or memantine (or both) was permitted in both studies provided this was at a stable dose for at least 18 weeks before randomization. Concomitant use of serotonergic antidepressant, antipsychotic (except clozapine or olanzapine), and sedative medications was also permitted at stable doses where clinically feasible. Patients were randomly assigned to receive LMTM at doses of 250, 150, or 8 mg/day in Study 015 and doses of 200 or 8 mg/day in Study TRx-237-005. Randomization in both studies was stratified according to geographical region, use of AD-labelled co-medications, and severity of AD. The co-primary outcomes in both studies were the Alzheimer’s Disease Assessment Scale–cognitive subscale, 11-item version (ADAS-cog_11_) and the Alzheimer’s Disease Co-operative Study–Activities of Daily Living, 23-item version (ADCS-ADL_23_) scale measured at baseline and every 3 months. MRI scans were undertaken at screening or baseline and at 3-month intervals. MRI acquisition protocol and parameters were standardized across sites and all data were centrally collected, quality-controlled, and analyzed blind by an imaging core lab (Bioclinica). Volumetric data were used to measure changes in whole brain volume (WBV, which provides a measure of the volume of grey and white matter), lateral ventricular volume (LVV), and other regional volumes including hippocampus, although only the global measures are reported in the present study. Baseline volumes were assessed using FreeSurfer 5.3, and volume change was assessed using Boundary Shift Integral and Tensor Based Morphometry for regional structures. Full details and study protocols for these two trials are available [35, 36].

### Plasma levels

Blood samples for assessment of parent MT, *N*-desmethyl MT, and total MT (sum of parent MT and a labile LMT conjugate) were collected from each patient on the first treatment visit (two samples: pre-dose and approximately 3.5 h after the dose) and at each subsequent on-treatment visit. The protocol specified that PK plasma sampling was to be conducted only at sites with adequate facilities (i.e., a refrigerated centrifuge and adequate capability to reliably freeze samples). Blinded analyses were conducted at the University of Aberdeen GLP Test Facility. MT levels in plasma were measured using liquid chromatography-tandem mass spectrometry. The MT moiety is ionized by the mass spectroscopy procedure and hence LMT and MT^+^ forms in plasma cannot be discriminated. The method was validated for use in the Phase III studies over the range 0.2 to 10 ng/ml. Extrapolated MT concentrations were available below the lower limit of quantitation (but above the lower limit of detection) in approximately 35% of the Day 1 patients randomized to the 8 mg/day dose. The method was validated for long-term sample storage of 162 days at –20°C. Data from samples stored for longer periods (up to 827 days) were found to produce somewhat higher levels systematically, but the conditional weighted residuals overlapped substantially, indicating that the overall precision was similar and that modifications to the pharmacokinetic model to take account of storage time were not necessary. Plasma samples from a total of 1,296 patients from the two Phase III studies in AD were available for analysis and, of these, 1,162 also had baseline and post-baseline efficacy outcome data available for analysis.

### Pharmacokinetic model

The development and validation of the pharmacokinetic model was undertaken independently by the Institute of Clinical Pharmacodynamics (ICPD, New York, US) and will be reported separately. It was developed in two stages based on data from four Phase I studies in healthy volunteers and in special populations of patients with renal or hepatic impairment covering doses ranging from 4 mg to 1000 mg of MT given as LMTM. The initial model accounted for all forms of MT measured. A simplified model restricted to parent MT concentration data was found to have equivalent accuracy for the description of results from a repeat-dose Phase I study of LMTM over the relevant dosing range. For reasons of computational convenience, the simplified model was used for the Phase III population PK analyses to permit per-subject parameter estimation. Application of the model to the observed parent MT concentrations from the Phase III studies was dependent on the availability of exact date and time of dosing relative to sample collection. This was recorded most accurately for samples collected in clinic on Day 1. Data from post-Day 1 visits were available, but the lack of precise timing of sample collection relative to last dose reduced their accuracy. Although the results were directionally comparable to the more precise Day 1 estimates, only the latter were used for the exposure-response analyses reported here based on the per-subject estimated steady state maximum concentration of the drug (C_max,ss_). Renal function, estimated using the Cockcroft-Gault equation for creatinine clearance [39], was a significant term in the model; age, weight, body mass index, albumin, and other factors were not significant.

### Statistical analyses

The exposure-response analyses were conducted independently by ICPD and verified by TauRx. As a first step in the analysis, the concentration-response data were reviewed for ADAS-cog_11_ decline at week 65 in all patients receiving LMTM at a dose of 8 mg/day using a sigmoid E_max_ (maximum response) model not requiring assumptions regarding exposure subgroups. The model was fitted using covariates geographical region, CDR at baseline, co-medication status, and baseline ADAS-cog_11_. A 90% bootstrap confidence interval was also calculated.

Having shown a concentration-dependent response for cognitive decline, a mixed-effects model for repeated measures analysis was used to characterize further the relationship between each efficacy endpoint and parent MT plasma exposure (steady state C_max,ss_) according to the following formula:

$$\begin{array}{cc} & \mathrm{pharmacological}\;\mathrm{activity}\;∼\;\mathrm{plasma}-\mathrm{level}\;\times \;\mathrm{visit}\;\\ & \;+\;\;\mathrm{co}-\mathrm{medication}-\mathrm{status}\;\;\times \;\;\mathrm{visit}\;+\;\mathrm{co}-\mathrm{medication}-\\ & \mathrm{status}\;\times \;\mathrm{plasma}-\mathrm{level}+\;\mathrm{geographical}-\mathrm{region}\;+\\ & \mathrm{CDR}-\mathrm{at}-\mathrm{baseline}\;+\;\mathrm{baseline}-\mathrm{score}\;\times \;\mathrm{visit} \end{array}$$

The following terms were categorical variables in the models: plasma exposure (C_max,ss_) (7 levels), visit (5 levels), co-medication status with AD drugs (2 levels), geographical region (2 levels), and CDR at baseline (3 levels). For the longitudinal analyses, plasma level was described by two levels (above or below threshold).

In addition to the exposure-response analyses described above, a further standard pharmacological concentration-response analysis was undertaken using the Hill equation [40] applied to the ADAS-cog_11_ and whole brain volume data to characterize the overall response profile quantitatively and to investigate the lower concentration limit of the treatment effect. The Hill equation was applied under the assumption of non-cooperativity and was applied with an additional linear term to permit trends occurring at high concentrations to be included in the model. The expanded Hill equation was applied to the data in the form:

$$\begin{array}{cc} & \mathrm{treatment}\;\mathrm{response}\\ & =E_{\min}\;-\;\left(E_{\max}\;\times \;\left(C_{\max,\;\mathrm{ss}}-C_{\min}\right)\right)/\\ & \;\;\;\;\;\left(EC_{50}\;+\;\left(C_{\max,\;\mathrm{ss}}-C_{\min}\right)\right)\;\\ & \;\;\;\;+\;\left(A\;\times \;\left(C_{\max,\;\mathrm{ss}}-C_{\min}\right)\right) \end{array}$$

where E_min_ and C_min_ are imposed zero values (assumed to be 11 ADAS-cog_11_ units or –30 cm^3^ for WBV at 0.29 ng/ml based on visual inspection of the data); E is the mean treatment response for any given C_max,ss_ subgroup; E_max_ is the maximum treatment response as estimated from a standard Hill equation without the additional linear term; EC_50_ is the C_max,ss_ at which the treatment response is 50% of the maximum response as estimated from the a standard Hill equation without the additional term; A × (C_max,ss_ – C_min_) is a further linear term in which A is estimated by the model to take account of the trends seen at high concentrations. This was fitted separately for LMTM added to approved symptomatic treatments and for those who took the drug as monotherapy, with parameters estimated using a non-linear least squares estimator.

In order to relate C_max,ss_ values to theoretical doses, a linear model was fitted to the mean plasma concentrations at the 8, 150, 200 and 250 mg/day doses:

$$\mathrm{estimated}\;\mathrm{dose}=22.22\;\times \;\left(C_{\max,\;\mathrm{ss}}-0.016\right)$$

where dose is in mg/day and C_max,ss_ is ng/ml units.

### Trial registration

The TRx-237-005 trial is registered at Clinicaltrials.gov (NCT01689233) and the European Union Clinical Trials Registry (21012-002847-28); and the TRx-237-015 trial registered as NCT01689246 and 2012-002866-11.

### Funding

The study was wholly sponsored by TauRx Therapeutics Ltd. (Singapore). The funder of the study took the lead in study design, undertaking the study, data interpretation, and initial drafting of the report.

## RESULTS

**Fig.1:**
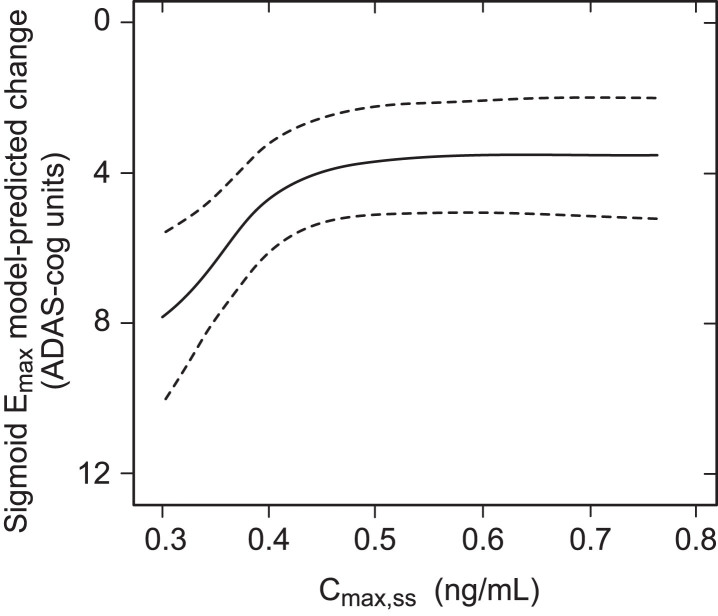
Sigmoid E_max_ analysis for ADAS-cog_11_ decline at week 65 with 90% bootstrap confidence intervals using estimated C_max,ss_ in patients receiving LMTM 8 mg/day.

The exposure-response relationship was first examined for decline on the ADAS-cog_11_ scale over 65 weeks in patients receiving the 8 mg/day dose using a sigmoid E_max_ analysis in which both the plasma concentration and cognitive decline data sets were included as continuous variables. As can be seen from Fig. 1, there is a clear relationship between cognitive decline and steady state plasma concentration of the drug at the 8 mg/day dose.

This implies that LMTM at a dose of 8 mg/day has a concentration-dependent effect on cognitive decline. In order to examine this relationship further, patients receiving LMTM at a dose of 8 mg/day were categorized on the basis of the C_max,ss_ into a low exposure group defined by the percentage of patients with first dose plasma levels below the level of quantitation of the assay (0.2 ng/ml); this corresponds to a modelled C_max,ss_ below 0.373 ng/ml. There were 208 such patients (35% of the 8 mg/day group). The remaining patients (N = 384) were split into three higher exposure group terciles to permit better visualization of trends (∼128 per group, 65% of patients receiving the 8 mg/day dose). Patients receiving doses in the range 150–250 mg/day were grouped according to dose (N = 187–329 per group) (Table 1).

**Table 1:** Parent MT C_max,ss_ for all patients with available plasma data according to either plasma C_max,ss_ subgroups (LMTM, 8 mg/day) or dose (LMTM, 150–250 mg/day)

Dose groups	*n* (%)	C_max,ss_ (ng/ml)	
		Mean (SD)	Range
8 mg/day – Group 1	208 (35%)	0.334 (0.0251)	0.257–0.373
8 mg/day – Group 2	127 (21%)	0.393 (0.0125)	0.373–0.414
8 mg/day – Group 3	129 (22%)	0.449 (0.0189)	0.415–0.478
8 mg/day – Group 4	128 (22%)	0.565 (0.0810)	0.479–0.812
150 mg/day	188 (100%)	7.820 (1.787)	5.099–18.611
200 mg/day	329 (100%)	10.126 (2.374)	6.557–21.291
250 mg/day	187 (100%)	12.573 (2.460)	8.833–21.188

**Fig.2:**
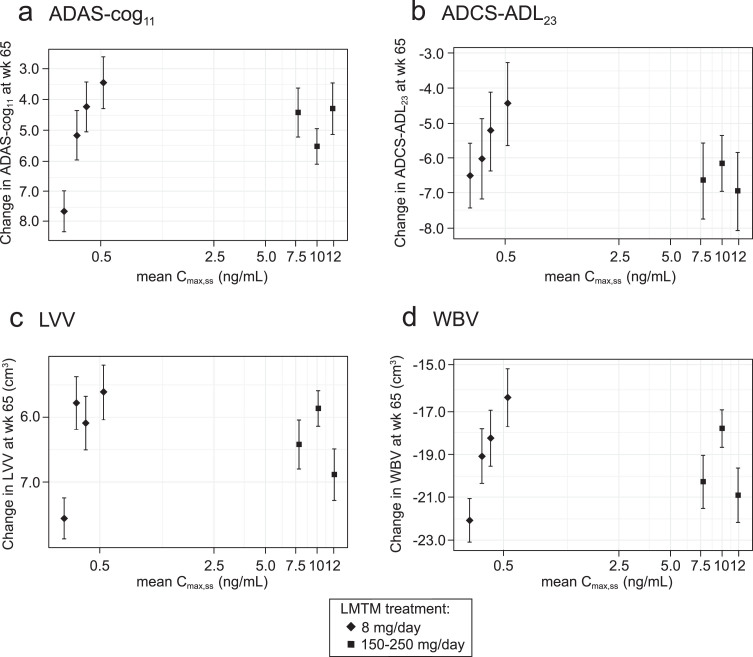
Model-derived least squares mean and standard error estimates of change over 65 weeks for clinical (a, b) and MRI volumetric endpoints (c, d) according to plasma concentration group (8 mg/day) or dose (150–250 mg/day) for all patients irrespective of co-medication status with AD-approved drugs.

Plotting of least squares mean and standard error estimates for change in ADAS-cog_11_, ADCS-ADL_23_, LVV, and WBV for these groups confirmed that clinical and volumetric outcomes all show similar concentration-dependent relationships at the 8 mg/day dose (Fig. 2). The effects seen at the substantially higher plasma levels associated with doses in the range 150–250 mg/day were no better than those seen at the 8 mg/day dose in patients having above-threshold plasma levels, consistent with the absence of an overall dose-response relationship as previously reported [35, 36].

### Effect of LMTM co-medication with AChEI and/or memantine

**Fig.3:**
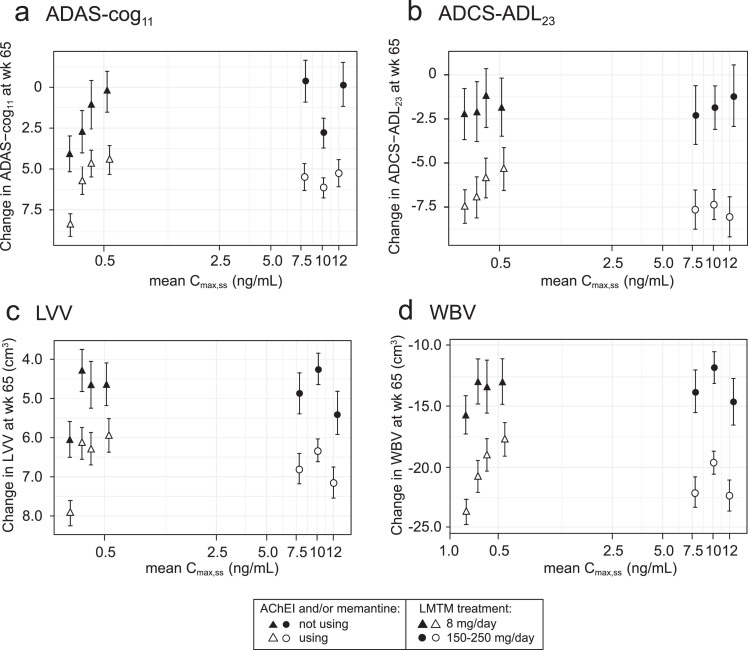
Model-derived least squares mean and standard error estimates of change over 65 weeks for clinical (a, b) and MRI volumetric endpoints (c, d) according to plasma concentration group (8 mg/day) or dose (150–250 mg/day) split by co-medication status with AD-approved drugs.

The exposure-response relationships for the same plasma C_max,ss_ subgroups (LMTM, 8 mg/day) or dose (LMTM, 150–250 mg/day) were examined separately according to co-medication status with approved AD treatments (LMTM alone or added to ongoing AChEI and/or memantine). As shown in Fig. 3, the concentration-response profiles are similar for LMTM as monotherapy and for add-on therapy, although the pharmacological activity is reduced for add-on therapy.

### Pharmacological analysis of concentration-dependent relationships for cognitive decline and brain atrophy

The concentration-dependent relationships for cognitive decline and loss of whole brain volume were explored further using the Hill equation which is commonly used in analyses of pharmacological activity [40]. The plasma C_max,ss_ subgroups (LMTM, 8 mg/day) or dose (LMTM, 150–250 mg/day) were used but, since part of the aim was to permit better estimation of the lower limit of the concentration-response relationship, the 208 patients with C_max,ss_ less than 0.373 ng/ml were split further into two equal groups of 104 patients (each representing 17% of the overall LMTM 8 mg/day group); the models were adjusted accordingly to 8 levels. Although plasma levels on Day 1 in this latter group were below the validated lower limit of quantitation of the assay, drug was still detectable and could be quantified by extrapolation from the assay calibration standards.

**Fig.4:**
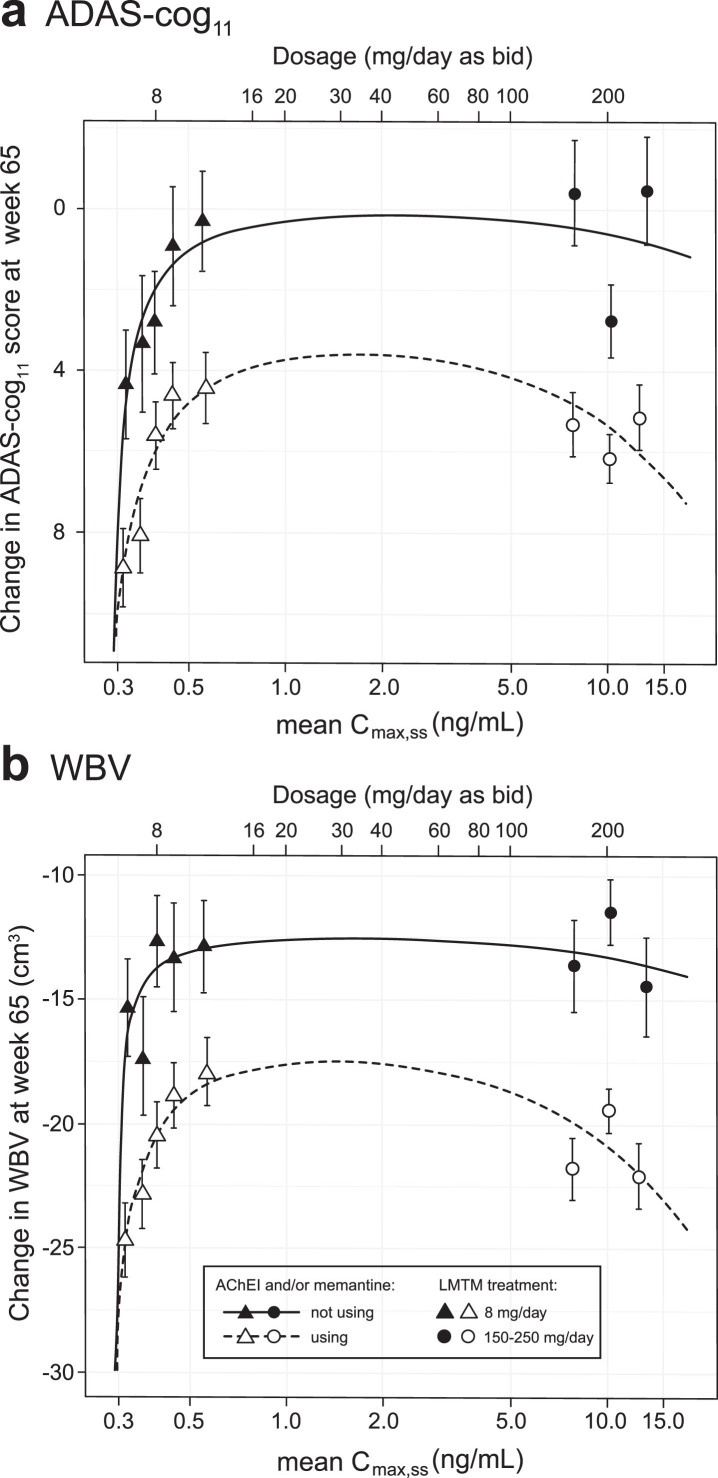
Hill equation analysis of pharmacological activity of LMTM on cognitive decline and brain atrophy over 65 weeks using model-derived least squares mean and standard error estimates of change over 65 weeks for clinical (a) and MRI volumetric (b) endpoints according to plasma concentration group (8 mg/day) or dose (150–250 mg/day) split by co-medication status with AD-approved drugs.

As can be seen from Fig. 4, the Hill equation provides a good fit to the concentration-dependent relationships for both cognitive decline and whole brain atrophy. For both outcomes, the response functions are consistent with a common lower limit of drug activity at approximately 0.29 ng/ml for both monotherapy and add-on therapy. Likewise, both clinical and volumetric responses are predicted to plateau above plasma levels corresponding to a theoretical dose of 16 mg/day. The maximum predicted pharmacological activity for LMTM as add-on therapy is about half that for monotherapy.

### Binary outcome analyses based on C_max,ss_ threshold of 0.373 ng/ml

We undertook further exploratory analyses to compare outcomes using patients with minimal drug exposure (i.e., having C_max,ss_ less than 0.373 ng/ml) as a proxy for placebo. Statistical comparisons of change in ADAS-cog_11_, ADCS-ADL_23_, LVV, and WBV are first shown for all patients regardless of co-medication status with approved AD drugs (Table 2A). The same comparisons restricted to patients taking the LMTM 8 mg/day dose are also shown in Table 2B. There were highly significant differences between the low and high plasma C_max,ss_ groups for all outcomes with the exception of ADCS-ADL_23_. This is true whether all patients are compared, or the analysis is restricted to patients receiving the 8 mg/day LMTM dose.

**Table 2:** Pharmacological activity analysis based on a threshold C_max,ss_ 0.373 ng/ml to define a proxy for placebo; modelled difference in change from baseline for the respective endpoints

	A) All patients, split by C_max,ss_ 0.373 ng/ml					B) Patients receiving LMTM, 8 mg/day, split by C_max,ss_ 0.373 ng/ml				
	Difference±SEM	CI	*p*	N_low-exposure_	N_high-exposure_	Difference±SEM	CI	*p*	N_low-exposure_	N_high-exposure_
ADAS-cog_11_	–2.66±0.69	–4.02, –1.30	<0.0001	193	969	–3.41±0.76	–4.89, –1.92	<0.0001	193	373
ADCS-ADL_23_	0.54±0.94	–1.30, 2.38	0.5634	192	967	1.22±1.01	–0.77, 3.21	0.2283	192	373
LVV (cm^3^)	–1.52±0.34	–2.18, –0.85	<0.0001	184	863	–1.76±0.38	–2.50, –1.01	<0.0001	184	335
WBV (cm^3^)	3.55±1.10	1.48, 5.61	0.0008	180	859	4.39±1.18	2.07, 6.71	0.0002	180	332

**Fig.5:**
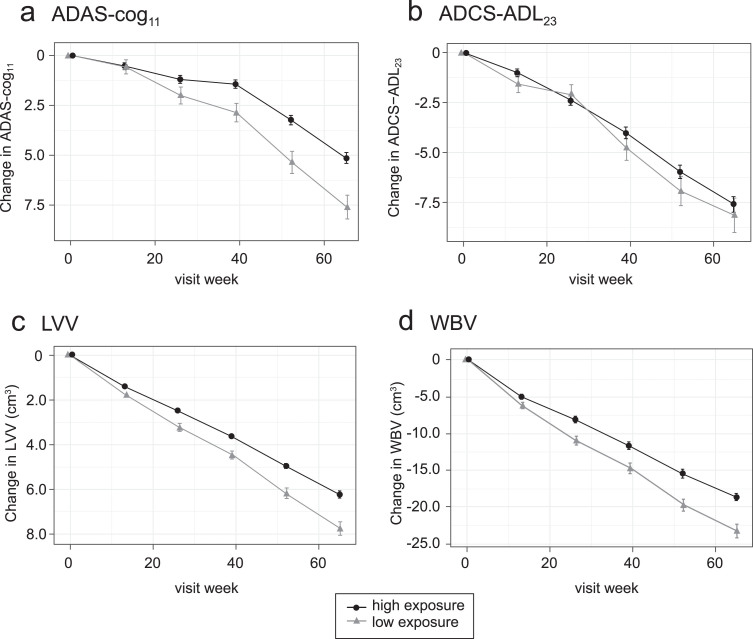
Comparison of primary clinical (a, b) and MRI volumetric (c, d) endpoints for all patients: categorized by C_max,ss_ above (“high exposure”) or below (“low exposure”) parent MT threshold of 0.373 ng/ml.

The corresponding longitudinal trajectories over 65 weeks according to C_max,ss_ above or below the threshold value of 0.373 ng/ml are shown in Fig. 5.

The analyses were repeated taking account of co-medication status with standard AD drugs for patients receiving the 8 mg/day dose. As shown in Table 3 and 3B, the same pattern of significant differences using the 0.373 ng/ml threshold was seen whether patients received LMTM at a dose of 8 mg/day as monotherapy or as add-on to AD-approved treatments.

**Table 3:** Comparison of patients receiving LMTM, 8 mg/day, with C_max,ss_ above or below the parent MT threshold of 0.373 ng/ml (A and B), and comparison of patients receiving LMTM, 8 mg/day as monotherapy, with C_max,ss_ above the parent MT threshold of 0.373 ng/ml with patients receiving the same dose as add-on to AD-approved treatments and having C_max,ss_ below the parent MT threshold of 0.373 ng/ml (C): modelled difference in change from baseline for the respective endpoints categorized according to AChEI and/or memantine use status at baseline

	A. LMTM, 8 mg/day, as monotherapy				
Difference±SEM	CI	*p*	N_low-exposure_	N_high-exposure_
ADAS-cog_11_	–2.60±1.16	–4.88, –0.33	0.0251	33	67
ADCS-ADL_23_	0.46±1.47	–2.43, 3.34	0.7552	32	67
LVV (cm^3^)	–1.60±0.46	–2.50, –0.70	0.0005	33	61
WBV (cm^3^)	2.76±1.66	–0.49, 6.01	0.0966	32	61
	B. LMTM, 8 mg/day, as add-on therapy				
	Difference±SEM	CI	*p*	N_low-exposure_	N_high-exposure_
ADAS-cog_11_	–3.52±0.78	–5.05, –2.00	<0.0001	160	306
ADCS-ADL_23_	1.32±1.04	–0.71, 3.36	0.2016	160	306
LVV (cm^3^)	–1.81±0.39	–2.56, –1.05	<0.0001	151	274
WBV (cm^3^)	4.69±1.21	2.32, 7.06	0.0001	148	271
	C. Comparison of LMTM, 8 mg/day, low C_max_ add-on versus high C_max_ monotherapy				
	Difference±SEM	CI	*p*-value	N_low-exposure_	N_high-exposure_
ADAS-cog_11_	–7.53±1.22	–9.93, –5.13	<0.0001	160	67
ADCS-ADL_23_	6.14±1.64	2.93, 9.34	0.0002	160	67
LVV (cm^3^)	–3.15±0.62	–4.37, –1.93	<0.0001	151	61
WBV (cm^3^)	11.54±1.87	7.88, 15.21	<0.0001	148	61

**Fig.6:**
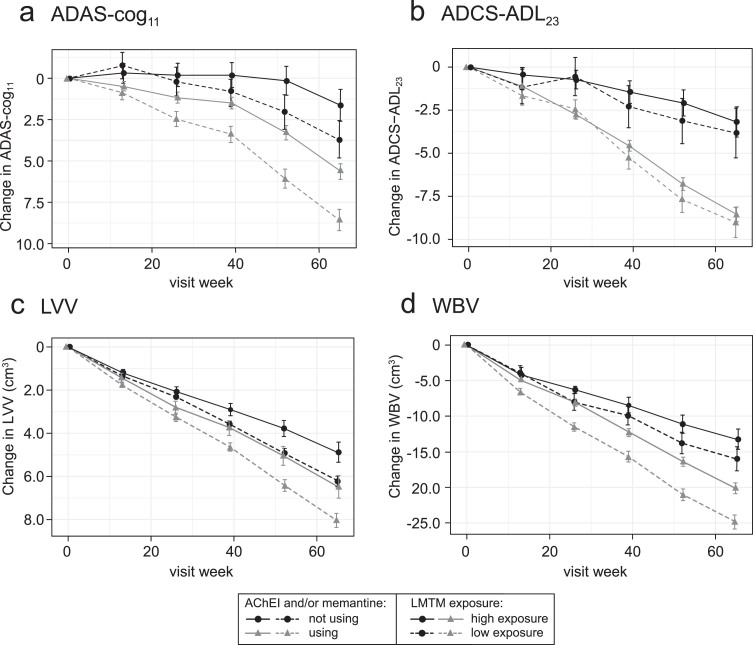
Comparison of primary clinical (a, b) and MRI volumetric (c, d) endpoints in patients receiving LMTM, 8 mg/day: categorized by C_max,ss_ above (“high exposure”) or below (“low exposure”) parent MT threshold of 0.373 ng/ml and AChEI and/or memantine use status.

The corresponding longitudinal trajectories over 65 weeks are illustrated below for ADAS-cog_11_, ADCS-ADL_23_, LVV, and WBV in Fig. 6.

Figure 4 suggests that the analysis using the 0.373 ng/ml threshold shown in Table 3A may underestimate the concentration-dependent difference in activity in patients receiving LMTM as monotherapy because of apparent activity even at the lowest MT concentrations measured. We have therefore undertaken a further analysis using patients taking LMTM as add-on who had subthreshold plasma levels as a common basis for comparison to examine the effect of LMTM as monotherapy in patients with above-threshold levels (Table 3C). As can be seen by comparing Tables 3B and 3C, the apparent exposure-dependent activity of LMTM as monotherapy is approximately double that seen for LMTM taken as add-on to standard treatments for all outcomes, consistent with the Hill equation analysis shown in Fig. 4.

### Influence of choice of threshold and intrinsic/extrinsic factors on plasma concentration

As noted, the classification threshold we have used in these analyses is based on the percentage of patients with plasma levels below the validated limit of the plasma assay on Day 1, and hence is independent of the outcome analyses. However, we have also examined the use of the median C_max,ss_ value as the cut-off for patients receiving the 8 mg/day dose as an alternative unbiased threshold. As shown in Supplementary Tables 1 and 2, the pattern of results is essentially the same, although the estimated pharmacological activities are smaller due to inclusion of more patients having some pharmacological activity in the low exposure arm.

We also investigated the influence of a number of intrinsic factors on C_max,ss_ classification: age, weight, body mass index, creatinine clearance, and serum albumin level as well as baseline clinical characteristics. Comparisons stratified by whether or not the patient achieved the C_max,ss_ threshold of 0.373 ng/ml at the 8 mg/day dose are provided in Supplementary Tables 3A and 3B and Supplementary Figure 1. Baseline clinical characteristics are also compared according to co-medication status with AD-approved drugs in Supplementary Table 3 C. While there is substantial overlap in the distributions of intrinsic factors, the most prominent trends are for subjects with above-threshold C_max,ss_ to be older and to have lower creatinine clearance than those with sub-threshold levels. Indeed creatinine clearance is the most important predictor of whether or not a patient achieves a parent MT C_max,ss_ above the threshold of 0.373 ng/ml at the 8 mg/day dose. The correlation between C_max,ss_ and creatinine clearance is shown in Supplementary Figure 2. As shown in Supplementary Figure 3A, the probability of achieving the C_max,ss_ threshold is nearly 100% at the lowest observed value of creatinine clearance (20 ml/min/1.73 m^2^) and falls consistently as creatinine clearance increases such that the probability is essentially zero at the highest observed creatinine clearance (106 ml/min/1.73 m^2^). Supplementary Figure 3B shows the percentage of patients expected to have plasma levels above the 0.373 ng/ml threshold irrespective of creatinine clearance over a range of total daily doses of LMTM given in twice daily divided doses.

We have also investigated the most commonly used non-AD-labelled concomitant medications as potential extrinsic factors influencing plasma concentration (Supplementary Table 4). In patients receiving the 8 mg/day dose, the only class having a significant effect is the antihypertensive/antiarrhythmic group of drugs, with more patients than expected by chance having subthreshold C_max,ss_ levels. This too may be linked to age and/or renal function, but the interaction has not been investigated further.

## DISCUSSION

We report the results of an exploratory *post hoc* population PK analysis based on estimated steady state plasma levels in 1,296 (1,162 with baseline and post-baseline clinical and neuroimaging data) out of 1,686 patients participating in either of two Phase III trials of LMTM in mild to moderate AD. The trials were designed and randomized to compare doses in the range 150–250 mg/day with a dose of 8 mg/day thought to be inactive and used to mask possible urinary discoloration. No differences were found between 8 mg/day and any of the high doses in the analyses of outcomes as randomized in either of the two Phase III studies [35, 36]. We now report that notwithstanding the lack of a dose-response for the doses tested, there is an exposure-response at an LMTM dose of 8 mg/day for change over 65 weeks on the clinical ADAS-cog_11_ and ADCS-ADL_23_ scales, and MRI measures of global brain atrophy (LVV and WBV) based on estimated steady state plasma concentration of parent MT. High doses producing plasma concentrations well above the threshold required for activity are not associated with any additional benefit.

The relationship between dose and pharmacological activity can be understood more readily within the overall concentration-response space that we have now been able to define. The lack of dose-response comparing 8 mg/day with high doses in the range 150–250 mg/day is attributed to two main factors: a steep concentration-response at the 8 mg/day dose and an apparent plateau at substantially higher concentrations. Over the range of steady state plasma concentrations 0.3–0.8 ng/ml, patients receiving the 8 mg/day dose differ systematically in the extent of decline on clinical and neuroimaging outcomes at 65 weeks. Differences in plasma concentration at this dose are shown to be driven primarily by renal function as measured by estimated creatinine clearance. Thus, patients with high creatinine clearance have relatively lower steady state plasma levels of the drug and vice versa. This is consistent with the fact that 70% of clearance of the drug has been shown to be via the kidneys and that patients with mild to moderate renal impairment have been confirmed to have higher plasma levels in an independent Phase I study in renally impaired subjects (unpublished observations).

Steady state concentrations in the range 0.3–0.8 ng/ml span a critical transition zone as regards pharmacological activity on clinical and neuroimaging endpoints. A recent study in minipigs using twice daily oral dosing of LMTM mimicking the regime and doses used in the Phase III trials has shown that the brain:plasma ratio is approximately 20 : 1 for the parent MT moiety at 2–4 h post-dose (unpublished observation). This compares with a ratio of 0.15 : 1 for MTC [20], which explains the large difference in dose required for pharmacological activity of LMTM and MTC. The brain: plasma ratio for LMTM also makes it possible to estimate the steady state concentrations of LMT in the brain as 6–16 ng/ml (0.021–0.056*μ*M) corresponding to the range of 0.3–0.8 ng/ml in plasma. We have reported recently that LMT blocks tau aggregation *in vitro* at a tau:LMT molar ratio of 1 : 0.1 [21]. Therefore, the brain concentration of aggregated tau at which LMT could theoretically have activity is less than ∼0.6 nmol/g. We have previously estimated the concentration of filamentous tau to be approximately 0.1 nmol/g in hippocampus (higher in entorhinal cortex and substantially lower in neocortex) in AD patients with MMSE score of approximately 20 units [41]. The concentration of oligomeric tau in AD brain is approximately 20% that of filamentous tau [42]. It is therefore plausible that the minimum plasma concentration required for pharmacological activity is determined substantially by the activity of LMT as a tau aggregation inhibitor.

In addition to the 8 mg/day dose having pharmacological activity in the majority of patients receiving it, the other factor consistent with the lack of a dose-response is that the pharmacological effects seen at plasma concentrations in the range 4–21 ng/ml are no greater than those seen in patients with a plasma concentration in the range 0.4–0.8 ng/ml. The estimates derived from the Hill equation analysis are consistent with treatment effects reaching a plateau at plasma concentrations in the range 0.9–4.1 ng/ml (corresponding to theoretical doses of 16–80 mg/day), although no confirmatory data are available in this intermediate range. It is common for drug responses to plateau above the concentrations/doses required for activity. What was unexpected for LMTM was that the threshold for pharmacological activity is so much lower than for MTC. We have previously reported that the concentration-response for a motor effect in an FTD tau transgenic mouse model with severe tau aggregation pathology shows a concentration-response up to a brain concentration of approximately 1*μ*M with no added benefit at higher concentrations [26]. The mechanisms responsible for this plateau are unknown, but could reflect a combination of limitations in neuronal capacity available for clearing the tau protein released by LMT from tau oligomers or filaments, homeostatic responses to the activating effects of LMTM [32] or negative effects on neuronal function at high concentrations. For example, the FDG-PET outcomes were found to be worse at the 200 mg/day dose than at the 8 mg/day dose in mild AD [35].

An important feature of the concentration-dependency relationships we report is the consistent difference in the pharmacological activity of LMTM alone or as add-on to approved symptomatic treatments for AD. These differences are seen at identical plasma concentrations and hence cannot be explained by pharmacokinetic differences in the absorption, distribution, or elimination of the drug. There are two possible explanations. The first might be that patients receiving symptomatic treatments have more severe disease and progress more rapidly than untreated patients, and that therefore smaller treatment effects are achievable. Patients receiving AD-approved treatments when they entered the trials were somewhat more impaired at baseline than those not receiving these treatments. However, the concentration-response analyses we have presented correct for the effects of baseline severity on rate of progression and outcome at 65 weeks, so this is unlikely to account for the difference. An alternative explanation, which seems more likely in the light of recent studies, is that the difference is neuropharmacological, since the same phenomenon can be reproduced in a well-characterized tau transgenic mouse model for AD [32]. Chronic pretreatment with either a cholinesterase inhibitor or memantine attenuates or eliminates many of the treatment effects seen when LMTM is given without such pretreatment. Tau-dependent LMTM treatment effects that are subject to interference by prior treatment with AD-approved drugs include reversal of behavioral deficits, effects on synaptic SNARE-complex proteins, and increase in brain mitochondrial Complex IV activity. Since these LMTM effects are not seen in wild-type mice, they are likely to represent secondary consequences of the primary action of LMTM on tau oligomers in synaptic terminals and in mitochondria. The MT moiety (given as MTC) has been reported to affect dopaminergic function in mouse models of Parkinson’s disease [43–45]. We have found that LMTM increases hippocampal acetylcholine levels in both tau transgenic and wild-type mice, and that this effect is also eliminated by pretreatment with a cholinesterase inhibitor [32]. LMTM may therefore have additional effects on neurotransmitter function which contribute to clinical activity and are not secondary to effects on tau pathology. The mechanism responsible for the reduction or elimination of LMTM effects in mice pretreated with AD-approved drugs appears to be part of a general homeostatic downregulation occurring in many neuronal systems in the brain that compensates for the activating effects of cholinesterase inhibitors and memantine. This has the overall effect of attenuating multiple neuropharmacological responses to LMTM, although the primary effect on tau aggregation pathology remains unaffected. The concentration-response profiles in patients receiving LMTM as an add-on are similar in form to those receiving LMTM alone, implying that LMTM has concentration-dependent activity both as monotherapy and as add-on to AD-approved treatments, albeit with the estimated maximum treatment effects seen clinically reduced by about half in the add-on group. This reduction in activity persists at high doses/concentrations of LMTM and is therefore not competitive.

Given that the 8 mg/day dose of LMTM appears to have pharmacological activity in the majority of patients receiving it, the only way to derive an estimate of the potential clinical treatment effects of LMTM from the data currently available to inform the design of a further placebo-controlled trial is to define a subthreshold patient group with minimal drug exposure as a proxy for placebo. The threshold we have used is based on the lower limit of quantitation of the plasma assay, namely 0.2 ng/ml. According to this criterion, 35% of patients receiving the 8 mg/day dose of LMTM had subthreshold plasma levels following their first dose on Day 1. We used this percentage to define patients with the least exposure to the drug with a steady state plasma concentration below a threshold of 0.373 ng/ml. Patients with plasma levels above this threshold have significantly less decline on ADAS-cog_11_, LVV, and WBV over 65 weeks than patients with sub-threshold plasma levels. Similar concentration-dependent differences are seen if the analyses were restricted to patients receiving 8 mg/day. Similar concentration-dependent differences are also seen when patients receiving LMTM alone or as add-on to symptomatic treatments are analyzed separately. These differences do not depend on the choice of a particular threshold, since the same pattern of results was seen when the median concentration at the 8 mg/day dose was used as the threshold. They also cannot be accounted for by measurable differences in severity, geography, or AD-approved co-medications since these variables are included in the analysis model. Further analyses including age and sex as covariates did not impact on the results.

The analyses we have presented provide the basis for the design of a confirmatory placebo-controlled trial in mild to moderate AD which is currently ongoing at sites in US, Canada, and EU. The trial aims to compare a dose of 16 mg/day with placebo. This is expected to produce an estimated mean plasma concentration of 0.88±0.18 ng/ml, which is close to the concentration required for the maximum predicted treatment effect of LMTM on cognitive and neuroimaging outcomes. At this dose, all patients are expected to have plasma concentrations above the 0.373 ng/ml threshold irrespective of renal function. Because of the larger apparent treatment effect in patients not receiving symptomatic treatments, the study is being conducted with LMTM as monotherapy in patients who have either discontinued or have not started taking symptomatic treatments for AD.

There are important limitations in the inferences which can be drawn from the present study. A *post-hoc* concentration-response analysis of the kind we have undertaken does not prove efficacy. It provides a means of determining how pharmacological activity is related to plasma concentration, and hence dose. However, pharmacological activity is not the same thing as efficacy. Efficacy can be established only by demonstrating a statistically significant effect on prespecified outcomes in a suitable randomized placebo-controlled trial. The analyses presented here have served the purpose of informing the design of the confirmatory placebo-controlled trial which is now ongoing. A further limitation is that the steady state plasma levels have been estimated from data available following a first in-clinic dose using a pharmacokinetic model developed and qualified in separate Phase I studies. This is a standard approach in population PK and exposure-response analyses. Although analyses incorporating actual steady-state plasma levels produced directionally similar results, the estimates are less accurate. Such analyses still require the use of a pharmacokinetic model to estimate drug exposure, and this in turn requires accurate recording of the time interval between the dose and the taking of the blood sample. Patients and their study partners are generally poor at reporting the time of taking the last dose prior to clinic attendance. In the study now ongoing, patients are required to take their treatment in clinic on the days when PK samples are collected in order to minimize this problem and to permit better estimation of actual steady state plasma levels. It should also be noted that the concentration-response relationships seen for cognitive and neuroimaging outcomes were much weaker for the ADCS-ADL outcome. This is consistent with the functional outcome having a larger standard deviation than the other outcomes, although the difference taking account of co-medication status with AD-approved drugs remains significant on this outcome.

### Conclusions and summary

The exposure threshold required for LMTM to have pharmacological activity on brain structure and function is much lower than anticipated based on an earlier study with MTC. This is due to substantially better brain uptake of the active LMT species. The lack of difference in outcomes between 8 mg/day and 150–250 mg/day we have reported previously can be understood as being due to a combination of activity of LMTM in the majority of patients receiving 8 mg/day and lack of additional activity at high doses. The exposure-dependent relationships are similar whether LMTM is taken alone or as add-on to approved AD treatments, although the maximum predicted effect as add-on therapy is about half that for LMTM taken alone irrespective of dose. A dose of 16 mg/day is predicted to be the minimum required to ensure that all patients have plasma concentrations in the range required for pharmacological activity and to maximize this activity.

## Supplementary Material

Supplementary Material
