# Supplementary material for: Concentration-Dependent Activity of Hydromethylthionine on Cognitive Decline and Brain Atrophy in Mild to Moderate Alzheimer’s Disease
Source: J Alzheimers Dis. 2019 Nov 26;72(3):931–46. doi: 10.3233/JAD-190772 (PMC6918900; doi:10.3233/JAD-190772)
Supplement: Supplementary Material [file jad-72-jad190772-s001.pdf]

Supplementary Material

Concentration-Dependent Activity of Hydromethylthionine on Cognitive Decline and Brain Atrophy in Mild to Moderate Alzheimer's Disease

Supplementary Table 1. Comparison of all patients with $C_{max,ss}$ above or below the median for 8 mg/day

	**A. All Patients (Split by 8 mg/day $C_{max,ss}$ median)**					**B. Patients Receiving LMTM 8 mg/day (Split by 8 mg/day $C_{max,ss}$ median)**				
	**Difference ± SEM**	**CI**	***p***	**N_{low}**	**N_{high}**	**Difference ± SEM**	**CI**	***p***	**N_{low}**	**N_{high}**
ADAS-cog	-1.91 ± 0.59	-3.06, -0.77	0.0011	281	881	-2.39 ± 0.72	-3.80, -0.98	0.0009	281	285
ADCS-ADL	0.21 ± 0.81	-1.38, 1.81	0.7924	281	878	1.12 ± 0.96	-0.77, 3.01	0.2448	281	284
LVV (cm^3)	-0.80 ± 0.30	-1.39, -0.21	0.0075	265	782	-0.99 ± 0.37	-1.72, -0.27	0.0074	265	254
WBV (cm^3)	2.47 ± 0.92	0.66, 4.28	0.0076	259	780	3.43 ± 1.14	1.20, 5.67	0.0026	259	253

Supplementary Table 2. Comparison of patients receiving LMTM 8 mg/day with $C_{max,ss}$ above or below the 8 mg/day median categorized by AChEI and/or memantine use status at baseline

	**A. LMTM 8 mg/day as Monotherapy (Split by 8 mg/day $C_{max,ss}$ median)**					**B. LMTM 8 mg/day as Add-on Therapy (Split by 8 mg/day $C_{max,ss}$ median)**				
	**Difference ± SEM**	**CI**	***p***	**N_{low}**	**N_{high}**	**Difference ± SEM**	**CI**	***p***	**N_{low}**	**N_{high}**
ADAS-cog	-2.75 ± 1.09	-4.90, -0.61	0.0119	51	49	-2.36 ± 0.74	-3.81, -0.91	0.0014	230	236
ADCS-ADL	0.52 ± 1.38	-2.18, 3.23	0.7045	50	49	1.30 ± 0.99	-0.63, 3.23	0.1869	231	235
LVV (cm^3)	-0.82 ± 0.44	-1.69, 0.05	0.0638	51	43	-1.06 ± 0.38	-1.80, -0.33	0.0047	214	211
WBV (cm^3)	1.07 ± 1.58	-2.02, 4.17	0.4972	50	43	4.02 ± 1.17	1.73, 6.31	0.0006	209	210

Supplementary Table 3A. Baseline demographic characteristics of patients receiving LMTM 8 mg/day split by $C_{max,ss}$ threshold of 0.373 ng/ml

	**LMTM 8 mg/day with $C_{max,ss}$ ≤ 0.373 ng/ml**	**LMTM 8 mg/day with $C_{max,ss}$ > 0.373 ng/ml**
Age, mean (sd)	66.26 (8.159)	73.11 (7.900)
Age, median, (iqr)	66 (60,72)	74 (68,79)
BMI (kg/m^2), mean (sd)	27.22 (9.804)	26.43 (4.422)
BMI (kg/m^2), median, (iqr)	26.00 (23.05, 29.13)	26.08 (23.48, 28.96)
Sex, Male, n (%)	117 (59%)	133 (35%)
Sex, Female, n (%)	83 (41%)	244 (65%)
Race		
American Indian or Alaska native, n (%)	0	1 (<1%)
Asian, n (%)	8 (4%)	25 (7%)
Black or African American, n (%)	5 (3%)	11 (3%)
Other, n (%)	3 (2%)	3 (1%)
White, n (%)	182 (92%)	333 (89%)

Supplementary Table 3B. Baseline clinical characteristics of patients receiving LMTM 8 mg/day split by $C_{max,ss}$ threshold of 0.373 ng/ml

	**LMTM 8 mg/day with $C_{max,ss}$ ≤ 0.373 ng/ml**	**LMTM 8 mg/day with $C_{max,ss}$ > 0.373 ng/ml**
MMSE, mean (sd)	21.34 (3.330)	21.13 (3.457)
ADAS-cog, mean (sd)	20.74 (9.400)	21.54 (9.410)
ADL, mean (sd)	64.11 (10.147)	62.64 (11.243)
WBV (cm^3), mean (sd)	989 (106)	934 (110)
LVV (cm^3), mean (sd)	50.6 (25.8)	49.6 (22.2)

Supplementary Table 3C. Baseline clinical characteristics of patients receiving LMTM 8 mg/day split by co-medication status with AD-approved drugs

	**A. LMTM 8 mg/day as Monotherapy**			**B. LMTM 8 mg/day as Add-on Therapy**			***p***
	**Mean ± SEM**	**SD**	**N**	**Mean ± SEM**	**SD**	**N**	
ADAS-cog	18.62 ± 0.98	9.83	100	21.91 ± 0.43	9.32	466	0.0025
ADL	65.81 ± 1.13	11.24	99	62.49 ± 0.50	10.88	466	0.0082
LVV (cm^3)	40.04 ± 2.16	20.92	94	52.34 ± 1.14	23.59	425	<0.0001
WBV (cm^3)	955.83 ± 10.57	101.97	93	954.34 ± 5.58	114.27	419	0.9006

Supplementary Figure 1. Box-and-whisker plots showing the distributions of various intrinsic factors, stratified by achievement of $C_{max,ss}$ threshold of 0.373 ng/ml in patients receiving LMTM 8 mg/day

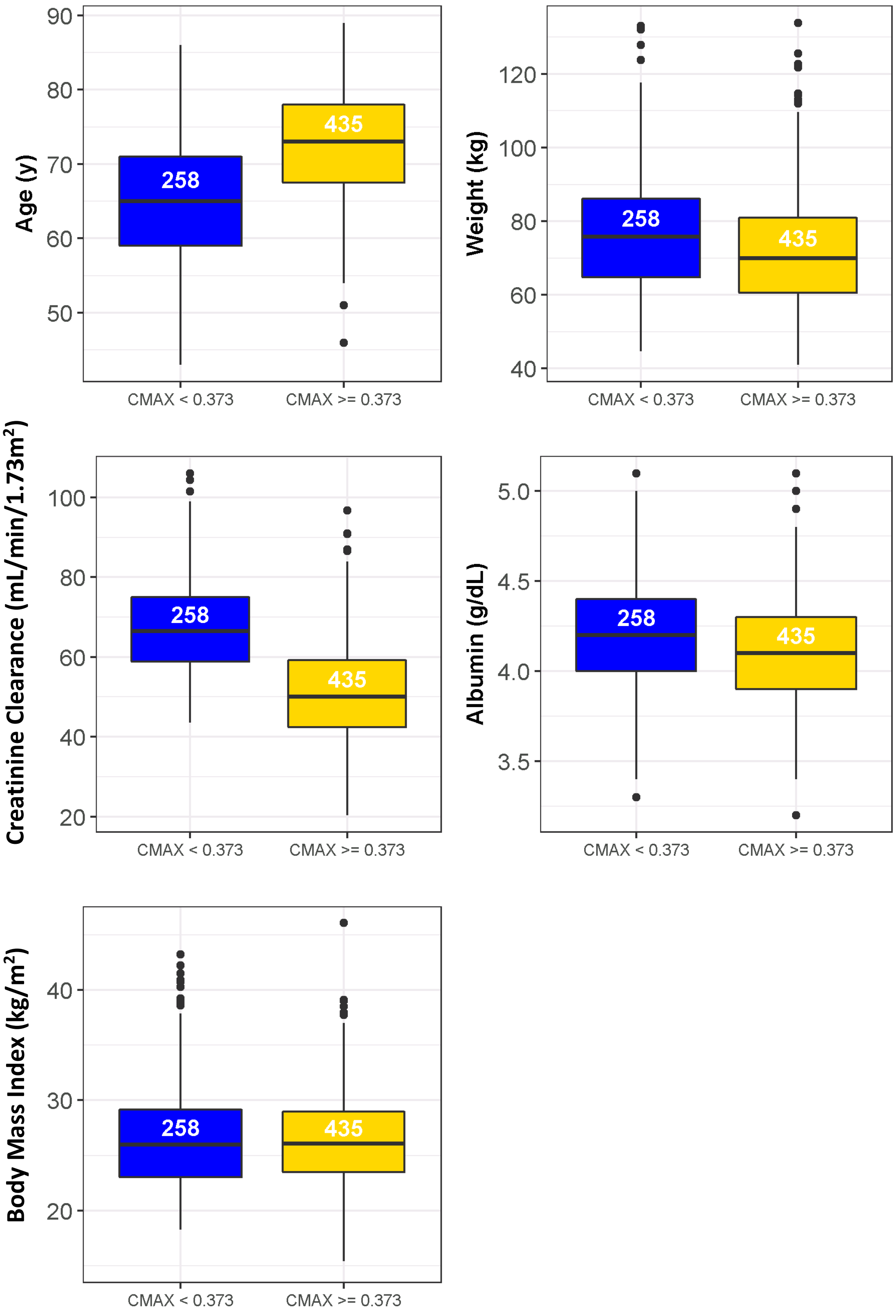

Supplementary Figure 2. Relationship between $C_{max,ss}$ and creatinine clearance by patient sex

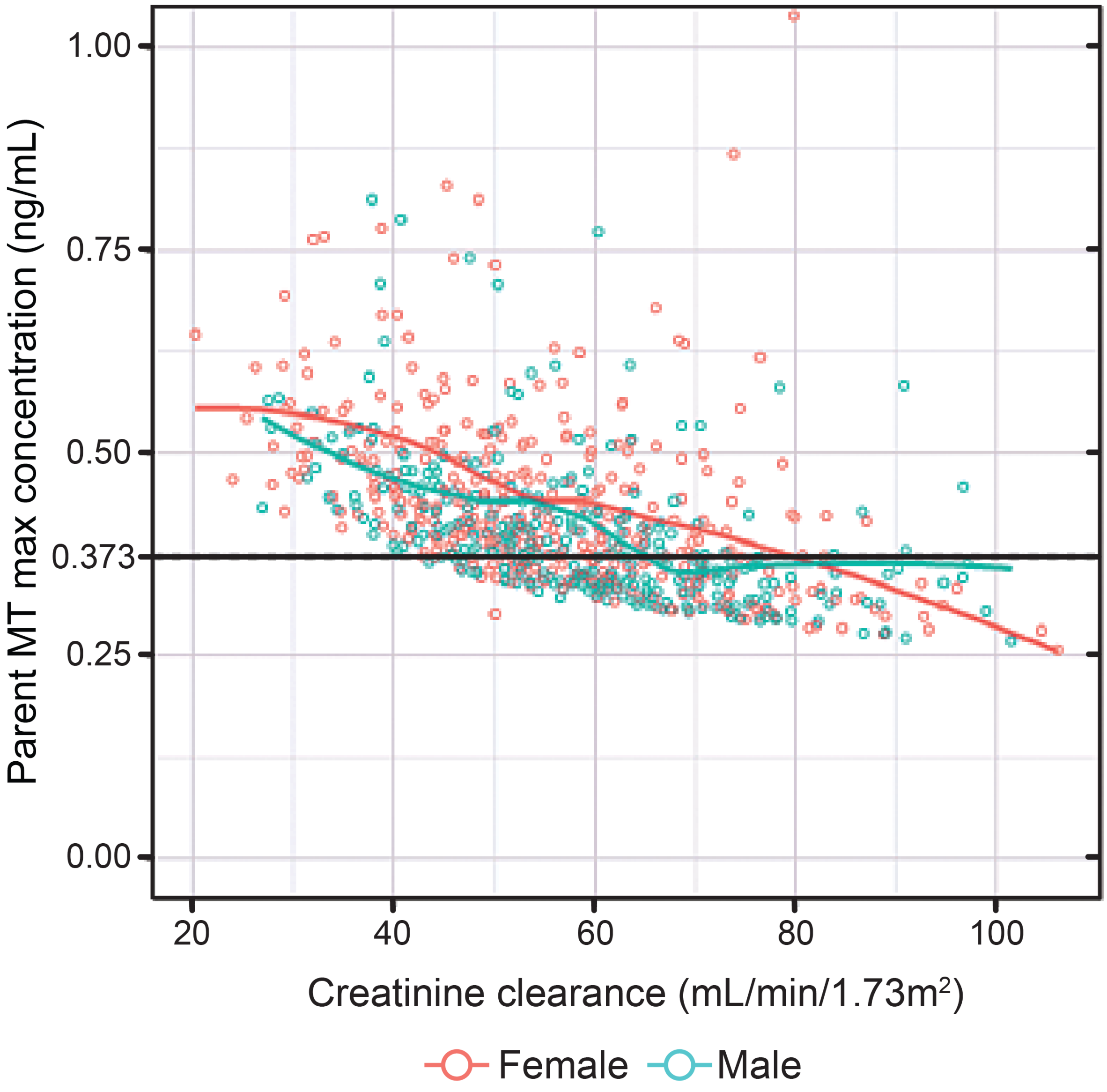

Supplementary Figure 3A. Probability of achieving the $C_{max,ss}$ threshold of 0.373 ng/mL at a dose of LMTM 8 mg/day versus creatinine clearance, overlaid on the observed data distribution

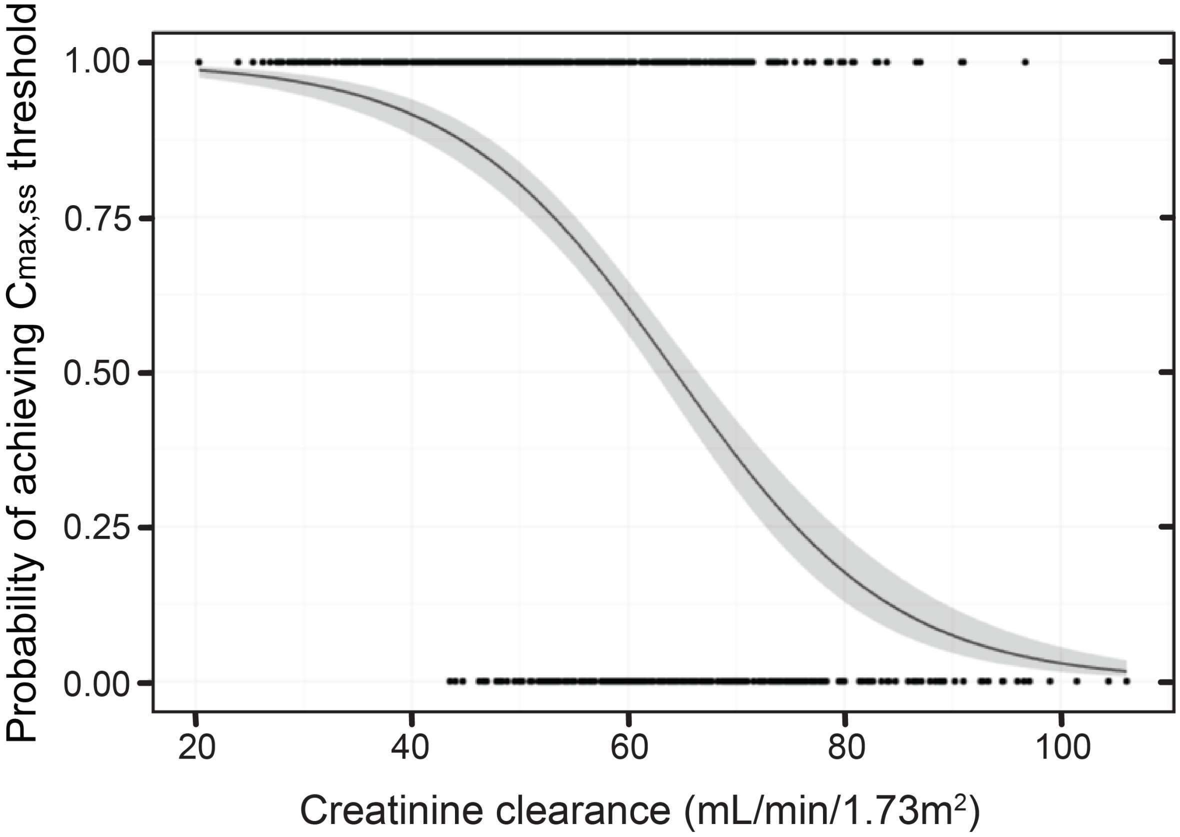

Supplementary Figure 3B. Expected percentage of patients with plasma levels above the 0.373 ng/mL threshold by total daily LMTM dose administered in divided doses b.i.d.

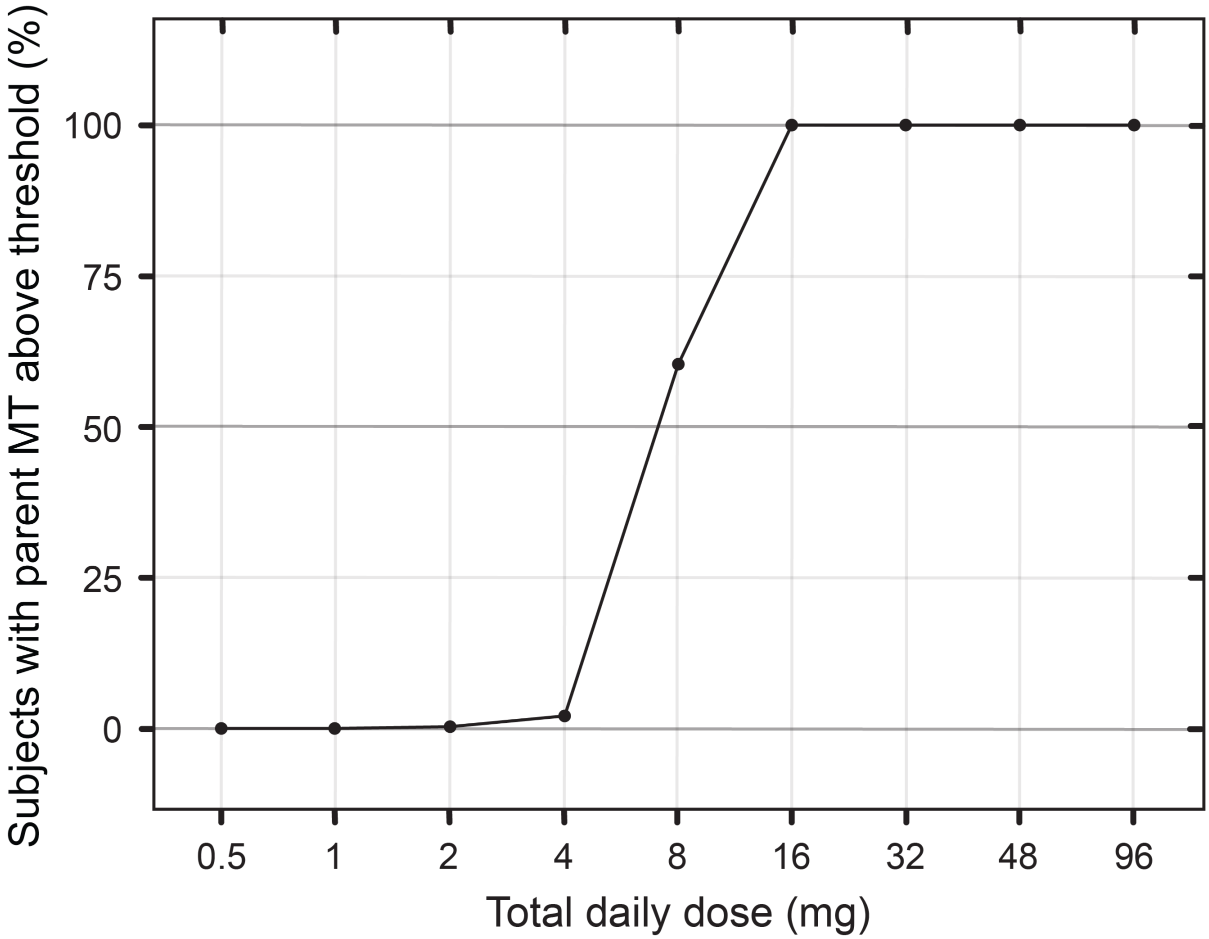

Supplementary Table 4. Summary of potential effect of non-AD-labelled concomitant medications (restricted to patients receiving LMTM 8 mg/day, with "high C_{max}" indicating $C_{max,ss}$ > 0.373 ng/ml)

WHO Drug ATC Class (Level 3)	N using (% high C_{max})	N not using (% high C_{max})	χ^2 statistic	*p*
Lipid modifying agents	282 (65.2%)	318 (66.0%)	0.01	0.907
Antithrombotic agents	239 (70.3%)	361 (62.6%)	3.44	0.064
Antihypertensive/antiarrhythmic drugs	200 (71.4%)	320 (60.6%)	7.26	0.007
Anti-inflammatory and antirheumatic products, non-steroids	78 (59.5%)	316 (67.3%)	2.45	0.117
Drugs for peptic ulcer and gastro-esophageal reflux disease	127 (66.9%)	309 (65.3%)	0.05	0.816
Thyroid preparations	63 (74.1%)	331 (64.3%)	2.72	0.099
Blood glucose lowering drugs, excluding insulins	77 (66.2%)	523 (65.6%)	0	1
